# Structure–Properties Relations for Polyamide 6, Part 1: Influence of the Thermal History during Compression Moulding on Deformation and Failure Kinetics

**DOI:** 10.3390/polym10070710

**Published:** 2018-06-27

**Authors:** Emanuele Parodi, Gerrit W. M. Peters, Leon E. Govaert

**Affiliations:** 1 Department of Mechanical Engineering, Materials Technology Institute, Eindhoven University of Technology, P.O. Box 513, 5600 MB Eindhoven, The Netherlands; Emanuele.parodi@maxxistce.nl (E.P.); g.w.m.peters@tue.nl (G.W.M.P.); 2 Faculty of Engineering Technology, University of Twente, P.O. Box 217, 7500 AE Enschede, The Netherlands

**Keywords:** polyamide 6, compression molding, polymorphism

## Abstract

The deformation and failure kinetics of polyamide 6 samples prepared by several thermal histories were investigated by tests at different temperatures and relative humidities. PA6 samples were produced in quiescent condition and multiple cooling procedure. A characterization was performed to investigate the effect of the different thermal histories and the effect of hydration on both structures and glass transition temperature. The mechanical properties were investigated by tensile and creep tests at different temperatures and relative humidity. In order to describe the experimental results, the Ree–Eyring equation, modified with the “apparent temperature”, was employed. In addition, the results of time-to-failure (creep tests) were described by the use of the “critical strain” concept. Eventually, a link between the Eyring theory and the structure evolution was made, i.e., a relation between the rate factors and the average lamellar thickness.

## 1. Introduction

The processing of polymer products is a widely discussed topic in polymer science. The solidification procedure is a crucial element in processing because of its influence on the structures which often affect the product performance. The mechanical properties of polymers are often correlated to their yield stress. Yield stress is defined as the stress at which the material deforms mainly plastically. In the case of glassy polymers, the solidification procedure affects mainly the thermodynamic state, i.e., ageing; it was found that ageing increases as the cooling rate during solidification decreases, consequently the yield stress increases [1]. The case of semi-crystalline polymers is more complicated; pressure and cooling rate can affect strongly the material morphology, i.e., crystallinity and lamellar thickness [2,3,4]. The influence of morphology (structures) on properties was studied by several authors [5,6], and most of them concluded that the mechanical properties are highly dependent on structures. The yield kinetics of i-PP solidified upon different processing was investigated by [7,8]; moreover, the results were described by the use of a model based on the Ree–Eyring equation. It was found that lamellar thickness (or crystal thickness) is a key parameter in order to model the yield kinetics of i-PP produced by different processing.

This work focuses mainly on the effect of processing on structures and the relations between structures and mechanical properties of polyamide 6 (PA6) tested at different temperatures and different relative humidity. PA6 crystallize, by melt processing, in two forms: (a) α-phase for slow cooling ($\dot{T}$< ≈8 °C), (b) γ-mesophase for intermediate cooling (≈8 °C < $\dot{T}$ < ≈100 °C); in case of quenching ($\dot{T}$ > ≈100 °C), a complete amorphous material can be obtained [9]. PA6 is hydrophilic [10], if exposed to a humid environment, it absorbs water. This is due to the polar character of its amide and carbonyl groups [11]. In dry conditions, the polar groups form hydrogen bonds between the polymer chains; these H-bonds give high strength to the material [12]. In case of hydration, part of the H-bonds between chains are broken and new H-bonds are made with the water molecules. This process, also known as plasticization, enhances the chain mobility and a decrease of glass transition temperature is obtained [13]. The hydration-induced depression of $T_{g}$ has a strong impact on mechanical properties [14,15,16,17] as well as crystallographic properties [18].

In this work, the mechanical properties (both tensile and creep test) of polyamide 6 samples prepared with different thermal histories, and conditioned at different relative humidities, are investigated. A semi-empirical model, based on the Ree-Eyring equation, was implemented to predict the yield kinetics and time-to-failure.

## 2. State-of-the-Art

Observing the yield kinetics (i.e., the yield stress as a function of applied strain rate) of several semi-crystalline polymers, two different stress-dependences can be seen. These two dependences are generally attributed to an intra-lamellar deformation mechanism further referred to as processes I and an inter-lamellar mechanism, further referred to as processes II [19,20,21]. To describe the rate- and temperature-dependence of yield stress obtained by tensile test at a constant strain rate, the Eyring’s activated flow theory [22], modified by Ree–Eyring [23], is used. In this theory, the two deformation processes are considered as independent and their stress contributions are additive. Consequently, the yield stress as a function of strain rate and temperature is calculated as follows:

(1) $$\sigma_{y}\left(\dot{\epsilon },T\right)=\frac{kT}{V_{I}^{*}}\;sinh^{-1}\left(\frac{\dot{\epsilon }}{{\dot{\epsilon }}_{0,I}}\;exp\left(\frac{\Delta U_{I}}{RT}\right)\right)+\frac{kT}{V_{II}^{*}}\;sinh^{-1}\left(\frac{\dot{\epsilon }}{{\dot{\epsilon }}_{0,II}}\;exp\left(\frac{\Delta U_{II}}{RT}\right)\right),$$

where ${\dot{\epsilon }}_{0,I}$, $\Delta U_{I}$ and $V_{I}^{*}$ are rate factor, activation energy and activation volume related to process I and ${\dot{\epsilon }}_{0,II}$, $\Delta U_{II}$ and $V_{II}^{*}$ are related to the process II. As mentioned in Section 1, the mechanical properties of PA6 are strongly dependent on the glass transition which depends on the sample conditioning, i.e., the hydration level. In fact, PA6 can absorb water, which acts as a plasticizer; in other words, the absorption of water lowers the glass transition and therefore the mechanical properties. Thus, in order to include the effect of relative humidity onto the Ree–Eyring equation, the “apparent” temperature was introduced. This temperature, as explained in [24], is based on the principle that a humidity-induced reduction in glass transition temperature is subsequently regarded as an “apparent” increase in the ambient temperature. Consequently, we express this concept as follows:

(2) $$\tilde{T}=T\;+\;\left(T_{g,dry}-T_{g,wet}\right),$$

where *T* is the actual experimental temperature, $T_{g,dry}$ is the glass transition temperature at the dry state and $T_{g,wet}$ is the $T_{g}$ after conditioning. Subsequently, Equation (1) is rewritten:

(3) $$\sigma_{y}\left(\dot{\epsilon },\tilde{T}\right)=\frac{k\tilde{T}}{V_{I}^{*}}\;sinh^{-1}\left(\frac{\dot{\epsilon }}{{\dot{\epsilon }}_{0,I}}\;exp\left(\frac{\Delta U_{I}}{R\tilde{T}}\right)\right)+\frac{k\tilde{T}}{V_{II}^{*}}\;sinh^{-1}\left(\frac{\dot{\epsilon }}{{\dot{\epsilon }}_{0,II}}\;exp\left(\frac{\Delta U_{II}}{R\tilde{T}}\right)\right).$$

Observing a creep test, i.e., a test with a constant applied load, three regimes can be found: (i) the primary creep in which the plastic flow rate decreases in time, (ii) the secondary creep where plastic flow rate is constant in time and (iii) the tertiary creep where plastic flow rate increases in time and finally failure occurs. Plotting, in a log–log scale plot, the plastic flow rate (${\dot{\epsilon }}_{pl}$) of the secondary regime as a function of time-to-failure, a linear trend with slope of −1 is found. This observation held for several polymers tested at different temperatures [25] and also different relative humidity [24]. Thus, the following relation is written:

(4) $${\dot{\epsilon }}_{pl}\left(\sigma \right)\cdot t_{f}\left(\sigma \right)=C,$$

where ${\dot{\epsilon }}_{pl}$ is the plastic flow rate in the secondary creep regime, $t_{f}$ is the time-to-failure, σ is the applied stress and *C* is the constant (−1). It was observed that the steady state reached by test at constant strain rate (i.e., yield stress) and the steady state achieved by test at constant load are identical [26]. Eventually, the relation between the strain rate dependence of yield stress and the load dependence of time-to-failure is described by the critical strain concept [27,28]:

(5) $$t_{f}\left(\sigma ,T\right)=\frac{\epsilon_{cr}}{{\dot{\epsilon }}_{pl}\left(\sigma ,T\right),}$$

where $\epsilon_{cr}$ is the critical strain that can be related to the amount of plastic deformation that the material would accumulate in the case in which the ${\dot{\epsilon }}_{pl}$ was constant all along the creep test.

## 3. Experimental

### 3.1. Materials

The material employed in this work was a polyamide 6 (Akulon K122) kindly provided by DSM ((Geleen, The Netherlands). This PA6 has a viscosity-average molar mass (Mv) of about 24.9 kg/mol.

### 3.2. Sample Preparation

Several different cooling procedures were performed in order to prepare sheets of 0.5 mm thickness with different structure parameters. After a drying procedure (1 night at 110 °C under vacuum), the pellets were placed in a “sandwich” consisting of two thick steel plates (about 3 mm), two thin aluminum foils (about 0.2 mm) and a 250 × 250 × 0.5 mm steel mold. The material was melted at 265 °C for 5 min, while a force of about 10 kN was applied. After this, different cooling procedures were applied (as summarised in Table 1):

- α-I, the hot press was switched off and the “sandwich” was left inside the hot press over night.
- α-II, the hot press was set to 180 °C, cooling was helped by a moderate flow of water, the “sandwich” was left inside during cooling and, once the set temperature was reached, it was removed after 5 min of isothermal.
- α-III, the same procedure of α-I was applied but an α-nucleating agent was added to the basic grade.
- γ-I, the “sandwich” was rapidly moved to a cold press set at 80 °C where the material was solidified in quiescent condition for 5 min.
- γ-II, the “sandwich” was rapidly moved to a cold press set at 110 °C where the material was solidified for 5 min.
- Q-I, the “sandwich” (only thin aluminum foils and mold) was rapidly moved to a bath of water with ice and salt (NaCl), water temperature around −14 °C.

According to the ISO527 type 1BA, dog-bone samples were prepared using a cutting die (main measures: width 5 mm, length 22 mm).

### 3.3. Sample Conditioning

In order to investigate the influence of hydration, the samples were stored at four different relative humidities, namely RH 0% (dry), RH 35%, RH 50% and RH 75%. For dry conditioning, samples were stored in a desiccators under vacuum at room temperature; for RH 50%, an environmental chamber was employed, while, in the case of RH 35% and RH 75%, two desiccators containing supersaturated salt solutions able to maintain a constant relative humidity in a close environment were employed. The supersaturated solutions were made of deionized water and two salts: sodium chloride and magnesium chloride hexahydrate for 75 and 35%, respectively.

### 3.4. Mechanical Tests

In order to perform uniaxial tensile and creep tests, a Zwick Unviversal Testing Machine (Ulm, Germany) provided with a 1 kN load-cell was employed. To control temperature and relative humidity, experiments were performed inside an environmental chamber. The tensile tests were repeated, at least, two times. Several conditions were investigated: a range of strain rates from $10^{-5}$ s^−1^ up to $3\;\times \;10^{-2}$ s^−1^, temperatures between 23 °C and 120 °C (dry) and relative humidity of 35, 50 and 75% (at 23 °C). A pre-load of 0.1 MPa was applied at a speed of 1 mm/min before each experiment. Creep was performed at three relative humidities: RH 35%, RH 50%, and RH 75%. The desired load was applied within 10 s, and kept constant up to failure. The time-to-failure was estimated as the time at which the strain reaches a fixed strain value of 40%, which was defined as strain at failure.

### 3.5. X-Ray Diffraction

**Figure 1:**
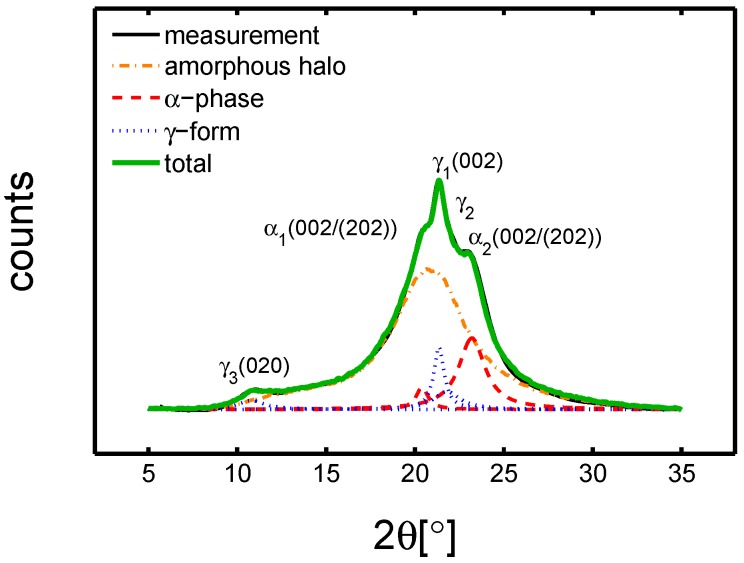
Example of wide angle x-ray diffraction (WAXD) pattern deconvolution analysis. The green line is the result of the deconvolution procedure, the orange line is the measured amorphous halo, and blue and red curves are the Lorentzian functions.

Wide and small angle X-ray measurements were taken by a Ganesha X-ray instrument (Copenhagen, Denmark) equipped with a GeniX-Cu ultra low divergence source (Copenhagen, Denmark) (*l* = 1.54 Å) and a Pilatus 300 K silicon pixel detector (487 × 619 pixels of 172 × 172 μm^2^). After normalization, the crystallinity was estimated by subtracting an amorphous halo (experimentally obtained) to the measured patterns. The degree of crystallinity is finally calculated by:

(6) $$\chi_{c}=\frac{T_{s}-A}{T_{s}},$$

where $T_{s}$ is the total scattered intensity and *I* is the scattering from the amorphous halo. Moreover, a deconvolution analysis was performed. This was obtained by fitting Lorentzian functions, in proximity of each characteristic reflection. Eventually, all the functions and the amorphous halo were summed to check the fidelity of the fitting routine (green markers in Figure 1). Thus, the relative quantities $\chi_{c,\alpha }$ and $\chi_{c,\gamma }$ were calculated by the following:

(7) $$\chi_{c,\alpha }=\left(\frac{A_{l}-A_{\gamma }}{A_{m}}\right)\;\;\;\;\;\;\;and\;\;\;\;\;\chi_{c,\gamma }=\left(\frac{A_{l}-A_{\alpha }}{A_{m}}\right),$$

where $A_{\alpha }$ and $A_{\gamma }$ are the total area of the Lorentzian functions for the α and γ peaks, $A_{m}$ is the total area of the measured pattern, and $A_{l}$ is the sum off all the Lorentzian functions (α and γ). An example is given in Figure 1. As far as the small angle X-ray scattering (SAXS) experiments are concerned, Lorentz [29] and thermal density fluctuation [30] correction were applied. Thus, the peak position of the SAXS pattern ($d^{*}$) is used to define the long period ($l_{b}$):

(8) $$L_{b}=\frac{2\pi }{d^{*}},$$

which is used to estimate the average lamellar thickness:

(9) $$l_{c}=\chi_{vol}\cdot l_{b},$$

where $l_{b}$ is the long period and $\chi_{vol}$ is the volumetric crystallinity, which is defined by the following:

(10) $$\chi_{vol}=\frac{\frac{\chi_{c}}{\rho_{c}}}{\frac{\chi_{c}}{\rho_{c}}+\frac{100-\chi }{\rho_{a}}},$$

where $\rho_{c}$ is the density of the crystal (1.21 g/cm^3^ for α-phase [31], 1.16 g/cm^3^ for γ-mesophase [31]), $\rho_{a}$ the density of the amorphous (1.09 g/cm^3^ [31]) and χ the mass crystallinity.

### 3.6. Dynamical Mechanical Thermal Analysis

Dynamical mechanical thermal analysis (DMTA) was employed to measure the glass transition temperature. The equipment was a TA instruments Q800 DMA (Asse, Belgium). The samples were films (rectangular shape) of about 5 mm width, 0.5 mm thickness. The sample were tested at a single frequency of 1 Hz and a temperature ramp (from −40 °C to 100 °C) with a heating rate of 3 °C/min. The $T_{g}$ was defined as the maximum in tan(δ).

## 4. Results and Discussion

### 4.1. Samples Characterization

**Figure 2:**
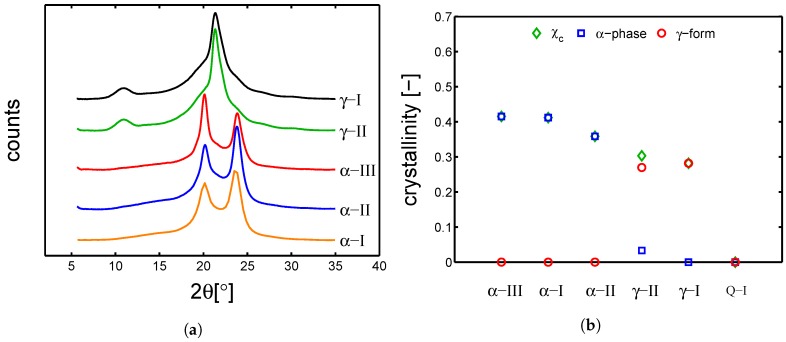
(**a**) wide angle X-ray diffraction integrated patterns; (**b**) deconvolution analysis of all the investigated samples with different thermal histories (dry state).

In order to understand the effect of different processing on structures, the first step of this study was a crystallographic characterization. It was performed by wide angle X-ray diffraction (WAXD) and small angle X-ray scattering (SAXS) on samples in the dry state. In Figure 2a,b, the integrated WAXD patterns and the results of deconvolution analysis are shown, respectively.

**Figure 3:**
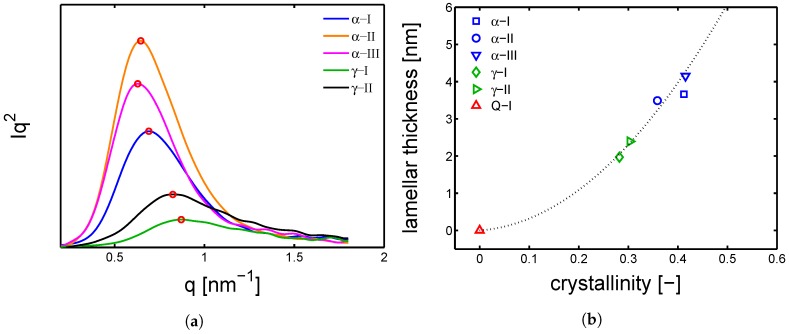
(**a**) small angle X-ray scattering integrated patterns in dry conditions; (**b**) lamellar thickness as a function of crystallinity in dry state. The dashed line is a guide to the eye.

In Figure 2a, the integrated patterns show that the samples γ-I and γ-I have crystallized in the γ-form, which is recognizable by the characteristic central peak at around 2θ 21°and the secondary peak at 2θ 10°; the obtained crystallinity is around 30% and by deconvolution it is possible to state that, only in the case of γ-II, a small fraction of α-phase is obtained, as shown in Figure 2b. The samples α-I, α-II and α-III showed the two characteristic peaks of α-phase, at about 2θ 20°and 2θ 24°; the obtained crystallinity is around 40% in the case of α-III and α-I, while a slightly lower crystallinity (about 35%) is obtained for α-II. These three α-samples have crystallized in pure α-phase. To estimate the average lamellar thickness, small angle X-ray were performed on the dry samples. Examples of SAXS pattern are given in Figure 3a; accordingly to the procedure described in Section 3.5, the average lamellar thickness is estimated and plotted as a function of crystallinity (see Figure 3b).

In Figure 3a, the integrated patterns are reported, the peak position (red markers) is translated to the long period by Equation (8). The relationship between $L_{c}$ and crystallinity is given in Figure 3b; the average lamellar thickness increases for increasing crystallinity with an asymptotic-like trend. It is remarked that, as stated in [32], crystallization of γ-mesophase at high under-cooling leads to the formation of non-lamellar morphology. Therefore, the values of $l_{c}$ for the γ-samples should be intended as crystal thickness rather than lamellar thickness.

### 4.2. Mechanical Properties

Next, the study can proceed with the investigation of the mechanical properties. This is initially done by tensile test at different temperatures, applying a range of strain rates (from $10^{-4}$ s^−1^ up to $3\;\times \;10^{-2}$ s^−1^) and the samples were kept in dry conditions. As a starting point for the mechanical properties’ investigation, only three cases will be investigated: α-I for the polymorph α, γ-I for the polymorph γ and Q-I for the complete amorphous material.

**Figure 4:**
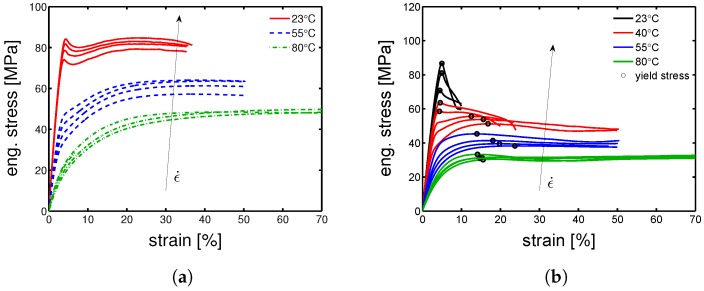
Stress-strain response at different temperatures and a range of strain rates for (**a**) α-I and (**b**) γ-I samples.

As expected, the stress–strain response increases as the strain rate increases for both α-I and γ-I; while it decreases as temperature increases (see Figure 4a,b). In the case of α-I at 23 °C, the stress–strain response shows a very clear double yielding (see Figure 4a). This occurs because the amorphous and crystalline domains yield at different strains; indeed, the yield at low strain range (about 5–10%) is regarded as the contribution of amorphous domains, and the yield at higher strain range (about 15–35%) is considered to be the contributions of crystalline regions. This effect can be simply proven by observing the effect of temperature (for a fixed strain rate) on the stress–strain response: the first yield, well visible at room temperature, tend to disappear as temperature increases and clearly disappear when the temperature is above $T_{g}$ (see Figure 4a green lines)—notwithstanding, also at temperatures higher than $T_{g}$, the double yielding would occur if very high strain rates were applied. The double yielding is less visible in the case of γ-I, which is likely due to a smaller contribution of the crystalline fraction, as demonstrated by the lower value of crystallinity estimated for γ-I (≈30%) compared with the one of α-I (≈40%). In order to study the yield kinetics, the yield stress is plotted as a function of the applied strain rate for different temperatures.

**Figure 5:**
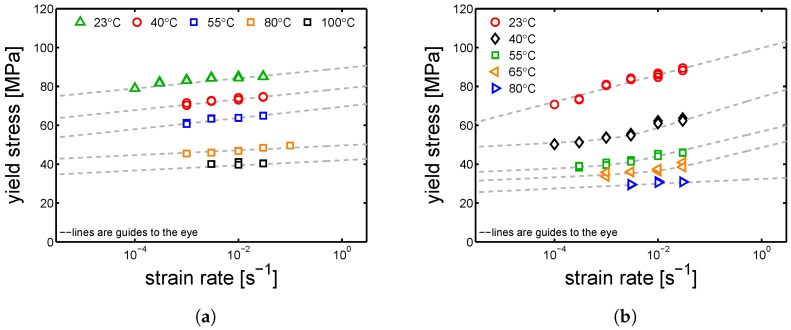
Yield kinetics (yield stress versus strain rate) at different temperatures for the (**a**) α-I and (**b**) γ-I samples. Lines are guides to the eye.

**Figure 6:**
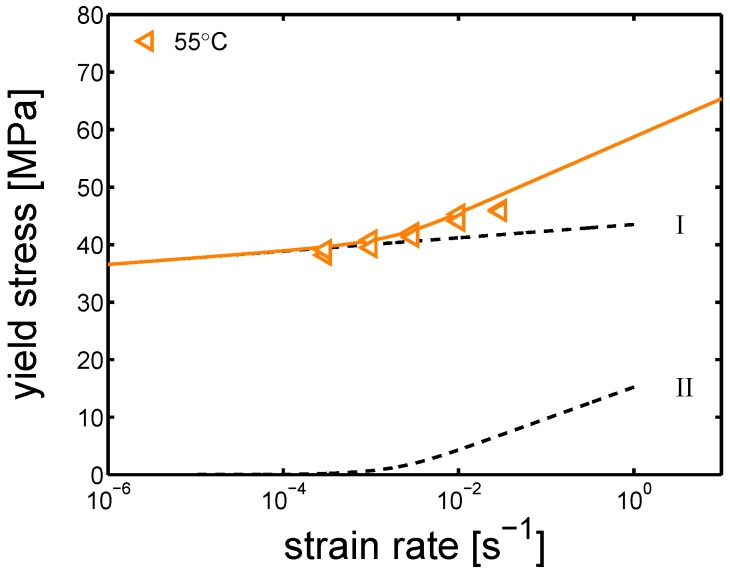
Example of two processes contributions. Yield stress versus strain rate at 55 °C; the black dashed lines are the two contributions separated (processes I and II).

In Figure 5a,b, the yield kinetics are shown; in both α-I and γ-I cases, two different strain rate dependences are observed, as mentioned in Section 2. A steep slope is observed at low temperatures and (or) high strain rates, while a rather flat one is displayed at high temperatures and (or) low strain rates. In Figure 6, a schematic decomposition of the two processes is proposed.

In order to describe the yield kinetics obtained experimentally, Equation (1) is used and the parameters in Table 2 and Table 3 were employed.

**Figure 7:**
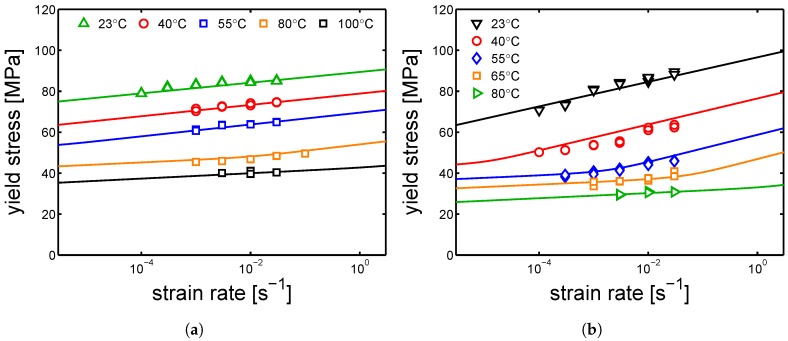
Stress–strain response at different temperatures (ranging from 23 °C to 100 °C) and a range of strain rate from $10^{-4}$ s^−1^ up to $3\;\times \;10^{-2}$ s^−1^ for (**a**) α-I and (**b**) γ-I samples. Lines are the results of Equation (1).

In Figure 7a,b, it is shown that the model can describe well the results at different temperatures for both α-I and γ-I. Moreover, it is remarkable that the activation energy and activation volume employed for process I are the same for both α-I and γ-I. The parameters of process II do not match between the two different polymorphs; a plausible reason could be found introducing the concept of amorphous constraint. Looking at the mobility scenario in a semi-crystalline polymer, it is known that the crystalline regions have the lowest mobility, whereas the amorphous region should have the highest mobility. However, an elevated presence of crystalline regions may constrain the amorphous regions, with a consequent decrease of mobility. Thus, a difference in crystallinity may affect also the state (mobility) of the amorphous regions.

### 4.3. Influence of Temperature

**Figure 8:**
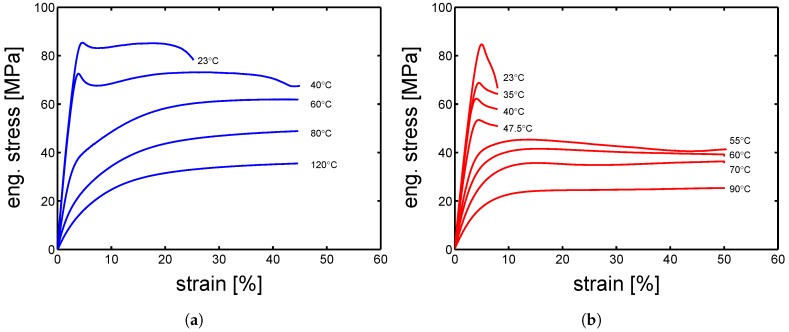
Stress-strain response at temperatures in a range from 23 to 120 °C, and strain rate of $10^{-2}$ s^−1^ for (**a**) α-I and (**b**) γ-I samples.

To obtain a large overview about the effect of temperature on the yield stress, several tensile tests were performed at $10^{-2}$ s^−1^ and several temperatures were tested (see Figure 8). Observing the results in Figure 8a, it is possible to notice a clear double yielding behavior at temperature lower than $T_{g}$, while increasing the testing temperature, the first yield (at low strains) tends to disappear. The α-I samples show a predominantly crystalline contribution to yield, this is due to a high crystallinity index and a relatively high lamella thickness. Figure 8b shows a very different picture; at low temperature, yield takes place at low strains (≈5%) and it moves to about 15% strain when the temperature is increased above 50 °C. Comparing the strain at yield for both α-I and γ-I samples, we observed a very similar strain for the yield related to the amorphous domains (about 5%), whereas the contribution of the crystalline domains takes place at quite a different strain range, ≈15% for γ-I and about 30% for α-I.

**Figure 9:**
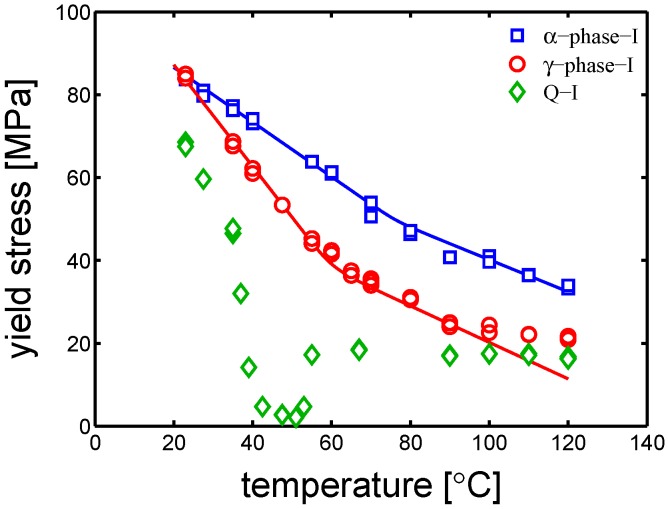
Yield stress versus testing temperature of samples tested with a strain rate of $10^{-2}$ s^−1^ and several temperatures. The lines are the results of Equation (1) in which the strain rate is a fixed value ($10^{-2}$ s^−1^) and the temperature is ranging from 23 to 120 °C.

Figure 9 shows that α-I, γ-I and Q-I samples have three very different temperature dependencies. For the green markers, related to Q-I samples, no description (solid line) is provided. In fact, looking at the trend of yield stress as a function of temperature, three regions are found: (i) at low temperature, yield stress decreases drastically as temperature increases; (ii) at about 50 °C, the yield stress reaches a minimum; (iii) after which the material starts cold crystallizing and the yield stress increases and reaches a plateau up to 120°. The Q-I stress–strain behavior variation is due to an evolution of the material morphology, which is time- and temperature-dependent. For this reason, the Q-I samples will be taken out of this study. As far as the blue and red markers (α-I and γ-I, respectively) is concerned, they show very similar yield stress at 23 °C, but, as temperature increases, γ-I yield stress decrease more rapidly than α-I. In these two cases, the model is applied: in the case of α-I, the experimental results are matching the description; in the case of γ-I, the description matches the experimental results up to 90 °C. After this temperature, the experimental yield stress flattens. As already explained in [24], an evolution of crystallinity and/or lamellar thickness takes place at high temperatures; this phenomenon, called “annealing”, is a time- temperature-activated process, it is governed by an enhancement of mobility that results in either cold crystallization and (or) lamellar thickening (perfectioning). Hence, higher crystallinity and (or) lamellar thickness results in an increase of yield stress, as shown in Figure 9. This effect is not observed in the case of α-I samples. It is hypothesized that “annealing” does not occur in α-I because of the already very high crystallinity and lamellar thickness obtained during processing.

The Ree–Eyring equation is applied with satisfactory results on the dry samples for both the two polymorphs, and two sets of parameters have been defined for the α-phase and γ-form, respectively; next, the influence of relative humidity and other thermal histories are investigated (see Table 1).

### 4.4. Influence of Humidity

As explained in Section 3.3, the samples were exposed to humid environments (relative humidity ranging from 35 to 75%) for a period long enough to allow the complete saturation. All the samples were conditioned at room temperature (23 °C). The absorbed water fraction was calculated by the following:

(11) $$W\%=(\frac{W_{i}-W_{0}}{W_{0}})\times 100,$$

where $W_{0}$ is the weight of the sample before conditioning and $W_{i}$ is the weight at the time $t_{i}$.

**Figure 10:**
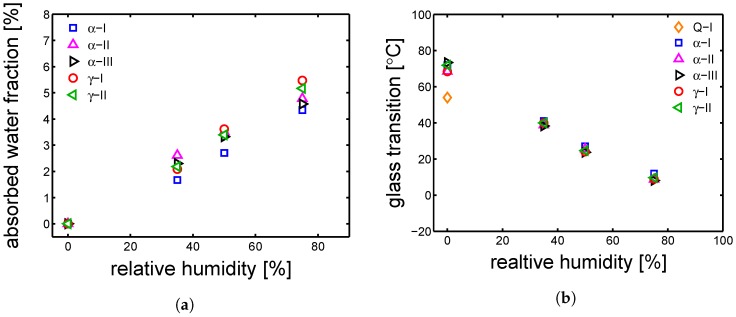
(**a**) absorbed water percentage at saturation in environments with different relative humidity at 23 °C. (**b**) glass transition temperature versus relative humidity.

**Figure 11:**
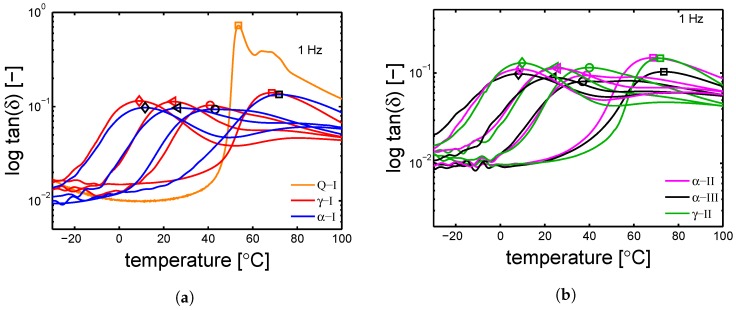
DMTA results, tan(δ) as a function of temperature for samples conditioned at different humidity; markers are the $T_{g}$ at (□) RH 0 %, (○) RH 35%, (◁) RH 50%, (◇) RH 75%. (**a**) samples α-I and γ-I; (**b**) α-II, α-III and γ-II.

**Figure 12:**
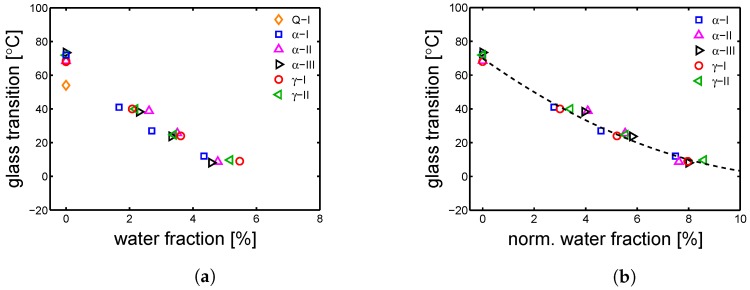
(**a**) glass transition temperatures (obtained by DMTA) as functions of the water fraction absorbed by the samples; (**b**) glass transition temperatures as functions of the normalized water fractions (line is a guide to the eye).

The absorbed water fraction is plotted as a function of relative humidity (HR%) (see Figure 10a). The saturation level is different between the the samples because of a difference in crystallinity; as mentioned in Section 1, water can be absorbed only by the amorphous regions (because of their mobility state). In Figure 10b, the measured glass transition temperatures are plotted as functions of the relative humidity; a monotonic decrease of glass transition temperature is found by increasing the RH%. The results of the thermal-mechanical characterization, performed by DMTA, are shown in Figure 11a,b. The details about this technique are given in Section 3.6. Figure 11a shows the results of DMTA for the samples Q-I, α-I and γ-I after conditioning at different RH%; the markers are the defined $T_{g}$s. The Q-I is presented only in a dry condition because, upon conditioning, it crystallizes and therefore it changes its state drastically. Figure 12a shows the glass transition temperatures as functions of absorbed water fractions. At RH 0% (dry condition), all the investigated samples show a maximum in glass transition temperature; increasing the absorbed water fraction, a monotonic decrease of $T_{g}$ is recorded. This is due to the plasticizing effect described in Section 1. Moreover, relying on the fact that only the amorphous region can absorb water, the glass transition temperature can be plotted as a function of normalized water fraction. The normalization is applied as follows:

(12) $$W_{n}\%=\frac{W\%}{1-\chi_{c}}\times 100,$$

where $\chi_{c}$ is the crystallinity by WAXD and W% is the water fraction estimated experimentally (see Equation (11)). The results are shown in Figure 12b. As proposed, $T_{g}$ follows a unique trend if plotted against a normalized water fraction. All of the $T_{g}$ are reported in Table 4.

**Figure 13:**
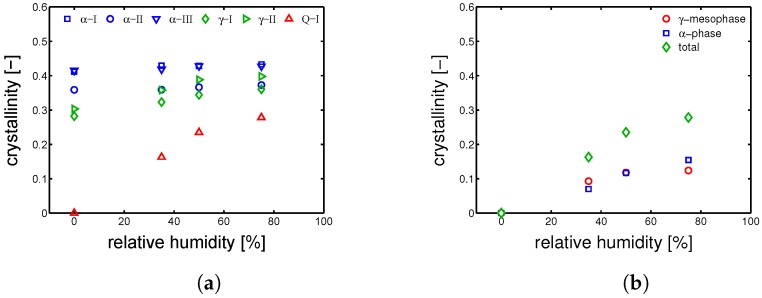
(**a**) overall crystallinity values as functions of relative humidity (conditioning performed at 23 °C); (**b**) evolution of the crystallographic phase contents as functions of relative humidity (conditioning performed at 23 °C) starting from Q-I (amorphous) sample.

As already mentioned in Section 1, the glass transition can drop at temperatures even below room temperature. In this case, the polymer chains acquire the sufficient mobility needed to cold crystallize and (or) thickening the pre-existing crystals. The crystallinity can be plotted as a function of relative humidity for different samples as shown in Figure 13a.

**Figure 14:**
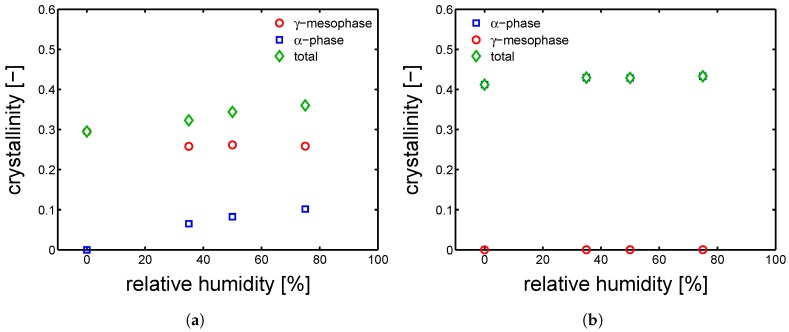
Evolution of the crystallographic phase contents as function of relative humidity (conditioning performed at 23 °C) starting from (**a**) γ-I and (**b**) α-I sample.

Figure 13a shows that hydration has an actual effect on the crystallographic properties of PA6. In details, the crystallinity of Q-I samples rapidly increases for increasing relative humidity, γ-samples show a modest increase of crystallinity and γ-samples show a rather constant crystallinity. In order to investigate the process of “cold crystallization” taking place in the Q-I samples, the deconvolution analysis of the WAXD patterns was performed; results are shown in Figure 13b. The samples Q-I, starting from a completely amorphous material, are exposed to three different relative humidities, always at room temperature. The lowest RH% (35%) leads to crystallization of γ-form and α-phase with a balance slightly shifted towards the γ-form; at 50 RH%, the total $\chi_{c}$ increases and the balance γ-α is perfectly even; at the highest investigated relative humidity (75%), the crystallinity increases even further and the balance γ-α is slightly shifted towards the α-phase. In the case of γ-samples, the starting material is already semi-crystalline with a rather high crystalline index, thus secondary crystallization, phase transition and (or) lamellar perfectioning (thickening) are expected. In Figure 14a, the results of deconvolution analysis for γ-I are shown. The $\chi_{c}$ increases along the whole range of relative humidity, in particular a slight decrease of γ-form and a substantial increase of α-phase is detected. This effect can be related to a partial transformation γ to α, followed by a secondary crystallization of α-phase. In the case of α-I (see Figure 14b), the deconvolution reveals that no transformation takes place and only the crystallinity seems to increase a little (probably within an experimental error).

**Figure 15:**
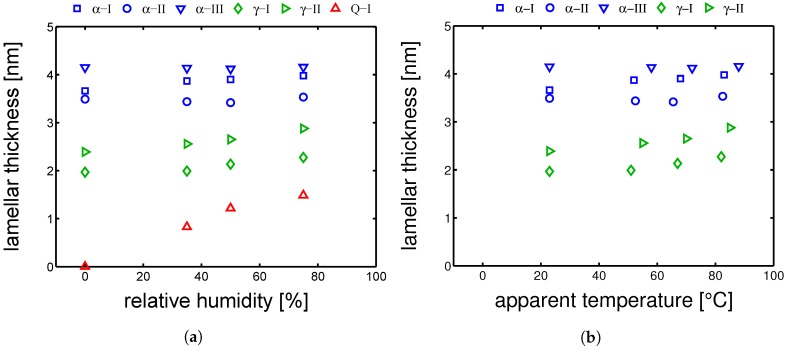
Lamellar thicknesses as functions of (**a**) relative humidity and (**b**) apparent temperature. All conditioning were performed at 23 °C.

In order to capture the lamellar thickness evolution upon hydration, SAXS experiments were performed (as explained in Section 3.5). In Figure 15a, the results of lamellar thickness are proposed as a function of relative humidity. A similar trend to $\chi_{c}$ versus RH% are found. The lamellar thickness of Q-I samples increase quickly with relative humidity; in addition, the $l_{c}$ of γ-samples increases although less rapidly than the amorphous samples, while the α-samples lamellar thickness are rather steady. These results can be plotted more intuitively as a function of the “apparent temperature”. In Figure 15b, the $l_{c}$ are plotted as a function of $\tilde{T}$, in this way, the increase of $l_{c}$ due to hydration can be easily regarded to a “cold crystallization” or “annealing” process, in which the samples are heated from the glassy state to a temperature above $T_{g}$.

**Figure 16:**
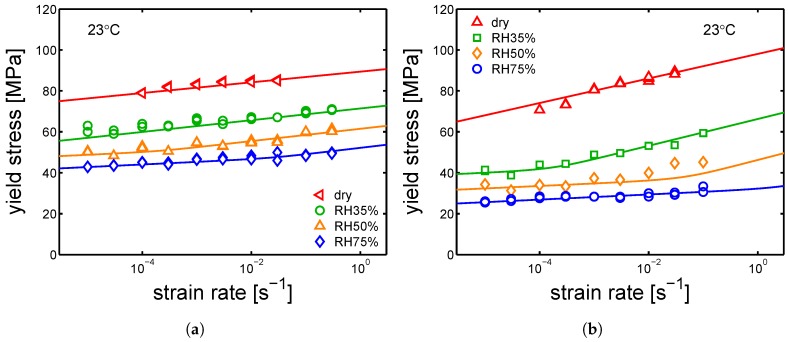
(**a**) yield stress as a function of strain rate for samples conditioned at different relative humidities, (**a**) α-I and (**b**) γ-I Testing temperature set at 23 °C.

Following this, the samples were tested by tensile test at different relative humidities and a range of strain rates ($10^{-5}$ s^−1^ up to $3\;\times \;10^{-2}$ s^−1^). As it was done for yield kinetics in dry conditions, the aim is to also describe the results of test at different relative humidities by the Ree–Eyring equation (see Section 2, Equation (3)) employing the two set of parameters defined for the α-phase and γ-form (see Table 2 and Table 3). Firstly, the model is applied to the samples α-I and γ-I (the results are shown in Figure 16a,b).

**Figure 17:**
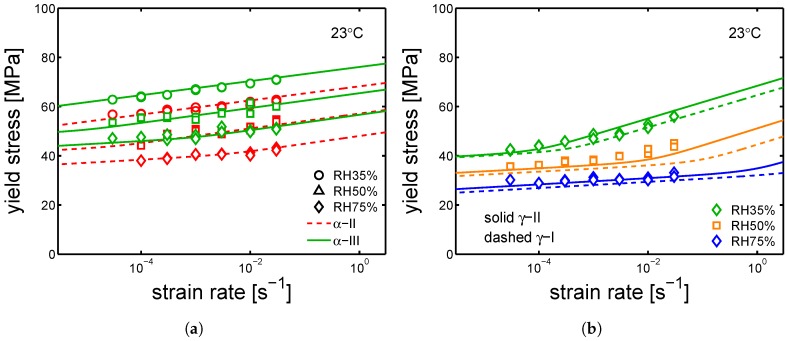
(**a**) yield stress as a function of strain rate for samples conditioned at different relative humidity, (**a**) α-II, α-III and (**b**) γ-II. Testing temperature set at 23 °C.

It is shown that Equation (3) describes the experimental results obtained at different conditions for two polymorphs of PA6. As shown in Table 2 and Table 3, the parameters employed for the α and γ polymorph differ mainly in the rate factors and the process II (inter-lamellar deformation). In Figure 17a,b, the yield kinetics of α-II, α-III and γ-II are shown, respectively. In order to describe these experimental results, the set of parameters used for α-I was employed for α-II and α-III, whereas the set used for γ-I was employed γ-II; in both cases, only the rate factors had to be changed. The parameters employed for these cases are listed in Table 5, Table 6 and Table 7.

### 4.5. Time-to-Failure

**Figure 18:**
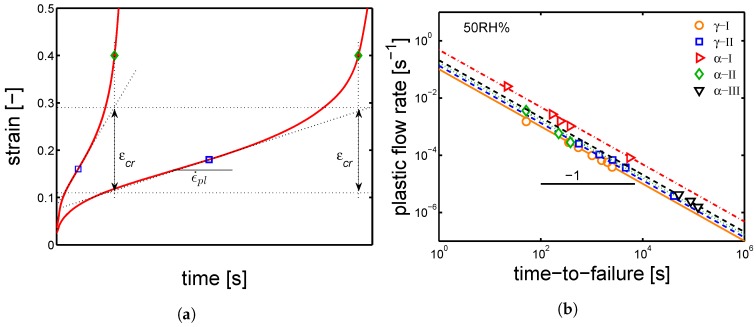
(**a**) examples of creep tests at constant applied load, the scheme shows the definition of ${\dot{\epsilon }}_{pl}$, $\epsilon_{cr}$ and $t_{f}$; (**b**) plastic flow rates as functions of time-to-failure for samples conditioned at RH 50% and 23 °C.

Next, the influence of relative humidity on the PA6 lifetime was investigated. Several creep tests were performed at 23 °C and relative humidity ranging from 35% to 75%. All of the five sample series were tested at different applied loads. Subsequently, the ${\dot{\epsilon }}_{pl}$ is estimated by the use of the Sherby–Dorn plot [33]; where the strain rate is plotted as a function of strain, and the ${\dot{\epsilon }}_{pl}$ is defined as the minimum of the obtained curve. Finally, all the ${\dot{\epsilon }}_{pl}$ are plotted as functions of the corresponding time-to-failure, as shown in Figure 18b. As mentioned in Section 2, the data plotted in a log–log graph show a slope of −1.

**Figure 19:**
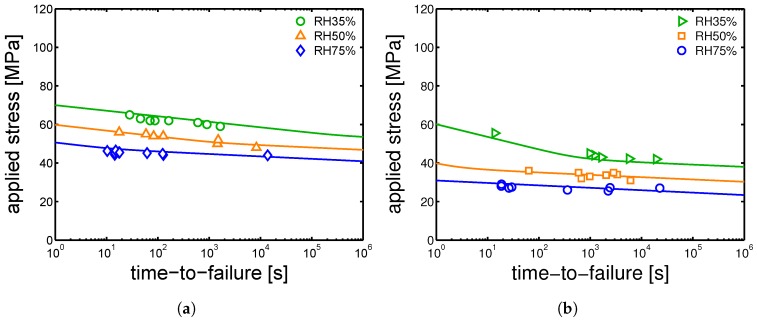
Creep tests for samples conditioned at RH 35%, RH 50%, RH 75% and 23 °C; applied loads as functions of time-to-failure for (**a**) α-I and (**b**) γ-I samples. Lines are the results of Equation (5).

By extrapolating to $t_{f}$ = 1 s, the results shown in Figure 18b, the $\epsilon_{cr}$ is estimated. Eventually, to describe the time-to-failure results obtained for the different samples series, the ${\dot{\epsilon }}_{pl}$ obtained by the Ree–Eyring equation (Equation (3)) modified to include the influence of relative humidity are combined with the $\epsilon_{cr}$ in Equation (5). In Figure 19a,b, the applied loads are plotted as functions of the time-to-failure. The lines are the results of Equation (5) and they are related to samples conditioned at different relative humidities (range from 35–75%) and 23 °C. The lines have the same stress dependence as the ones shown in Figure 16a,b but the slopes have opposite signs.

**Figure 20:**
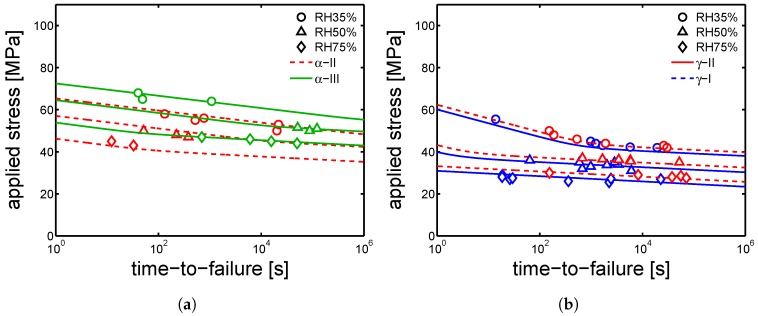
Creep tests for samples conditioned at RH 35%, RH 50% and RH75 % and 23 °C; applied loads as functions of time-to-failure for (**a**) α-II, α-III, (**b**) γ-I and γ-II samples. Lines are the results of Equation (5).

Figure 19a,b shows that a satisfactory prediction of time-to-failure is achieved for samples α-I and γ-I employing the set of parameters reported in Table 2 and Table 3 for samples α-I and γ-I, respectively. By the use of the parameters listed in Table 5, Table 6 and Table 7, also samples α-II, α-III and γ-II are described by Equation (5); the results are shown in Figure 20a,b.

### 4.6. Structure–Properties Relations

For all the investigated PA6 samples, the temperature, relative humidity and stress dependent deformation kinetics were captured by the Ree–Eyring theory. To apply this theory, the characteristic parameters were defined, namely the activation volume ($V^{*}$), the activation energy (ΔU) and the rate factors (${\dot{\epsilon }}_{0}$). As explained in Section 4.2, PA6 shows two strain rate dependences that are related to two deformation mechanisms: an intra-lamellar deformation mechanism (also called process I) and an inter-lamellar mechanism (also called process II). As shown in Equation (1), each process needs one set of parameters.

**Figure 21:**
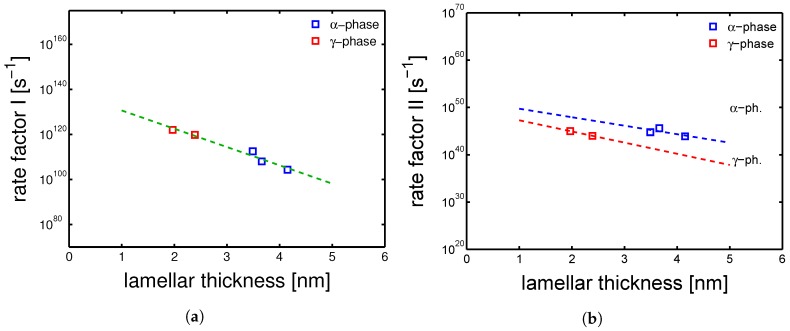
(**a**) rate factor I and (**b**) rate factor II as functions of lamellar thickness. Lines are guides to the eye.

The analysis has led to the conclusion that: (i) for process I, identical activation volume and activation energy can be used for all of the sample type, a part for the quenched samples whose structures, as explained in Section 4.3, are very dependent on temperature and relative humidity; (ii) for process II, $V^{*}$ and ΔU are different for the two different polymorphs, i.e., α-phase and γ-mesophase; (iii) the rate factors were varied for each samples series, for both process I and process II. About the different $V^{*}$ and ΔU determined for the two crystallographic phases in the case of process II, the author can only hypothesize that the reason might be in the different constriction level of the amorphous phase. In fact, as proposed in a previous study [24], process II is associated to the deformation of the amorphous phase. About the rate factors (${\dot{\epsilon }}_{0,I}$ and ${\dot{\epsilon }}_{0,II}$), a rather clear correlation between the lamellar thickness ($l_{c}$) was found (see Figure 21a,b).

Figure 21a shows the relation between the rate factor I and the lamellar thickness; plotting the logarithm of ${\dot{\epsilon }}_{0,I}$ as functions of lamellar thickness for both α and γ samples, a linear trend is found. In the case of ${\dot{\epsilon }}_{0,II}$, two trends are found: one related to the α-samples and one for the γ-samples (see Figure 21b). Remember that also the $V^{*}$ and the ΔU are different for process II, therefore there is no reason to expect that the trend of ${\dot{\epsilon }}_{0,II}$ for alpha matches the one for γ samples. This relation between the rate factors and the lamellar thickness was also proposed by other authors in the case of i-PP (isotactic polypropylene) [7,8]. The funding shown in Figure 21a,b are crucial for the prediction of the deformation kinetics and time-to-failure of PA6 processed with different histories. It is important to remark that, in the case of “real-life” applications, products are designed to work upon loads largely below the yield stress, hardly in dry conditions and often at high temperatures. Thus, the most frequent failure mode would be governed by intra-lamellar deformation, i.e., process I; consequently, the prediction would be governed by a $V^{*}$ and ΔU, which are not dependent on the crystallographic phase.

## 5. Conclusions

In this work, the influence of thermal history on the structure of PA6 was investigated in regards to the mechanical properties (short and long term failure) of samples tested at different temperature and relative humidity. The investigation has led to several conclusions:

- the thermal history, i.e., the solidification procedure, has a crucial effect on the polymorphism and subsequently on the stress–strain response. By observing the three extreme cases (α-I, γ-I and Q-I), differences in stain rate- and temperature-dependence of the stress–strain response were found. The α-I samples has shown the least dependence on strain rate (see Figure 7a) and temperature (see Figure 9), while Q-I resulted in the most dependence on strain rate and temperature, and γ-I has shown a moderate dependence on strain rate and temperature.
- by quenching from the molten state, completely amorphous samples (Q-I) were obtained; these samples are extremely sensitive to temperature and relative humidity. After heating a Q-I sample above ≈50 °C, cold crystallization is obtained. Note that this phenomenon is time-temperature-dependent, therefore according to the temperature and time span of testing, different stress–strain response is obtained. In addition, in case of exposition to moisture, cold crystallization occurs even at relative humidity of 35%.
- the exposition to moisture induced secondary crystallization, thickening of lamella and phase transformation (γ to α) for the γ-I and γ-II samples (see Figure 14a and Figure 15a).
- the Ree–Eyring equation was successfully applied to describe the yield kinetics of samples α-I and γ-I. This was possible by employing two set of parameters, with identical $V^{*}$ and ΔU for the process I and different $V^{*}$ and ΔU for the process II.
- a modification of the Ree–Eyring equation to include the effect of relative humidity on the glass transition was applied successfully on all the investigated samples (a part for the quenched ones). Moreover, by the use of the critical strain concept, the time-to-failure of samples conditioned at different relative humidity were described.
- a correlation between the rate factors and the lamellar thickness was found. For a given activation volume and activation energy, the corresponding rate factor is dependent on the lamellar thickness ($l_{c}$).
- the effects of relative humidity on the crystallographic properties (i.e., lamellar thickness, crystallinity and crystal phase) are considered negligible at the scope of description of mechanical properties.

## Figures and Tables

**Table 1:** Cooling protocols.

Sample	Method
α-I	slow cooled $\dot{T}$ ≈ 0.5 °C/s
α-II	isothermal at 180 °C
α-III	slow cooled $\dot{T}$ ≈ 0.5 °C/s—with nucleating agent
γ-I	isothermal at 80 °C
γ-II	isothermal at 110 °C

**Table 2:** Eyring parameters for α-I.

	$V^{*}$ [m^3^]	ΔU [J mol^−1^]	${\dot{\epsilon }}_{0}$ [s^−1^]
*I*	9e-27	1e6	1e108
II	6e-27	3.2e5	4e45

**Table 3:** Eyring parameters for γ-I.

	$V^{*}$ [m^3^]	ΔU [J mol^−1^]	${\dot{\epsilon }}_{0}$ [s^−1^]
*I*	9e-27	1e6	1e122
II	1.9e-27	3e5	2e45

**Table 4:** Glass transition temperature [°C].

Sample	Dry	35 RH%	50 RH%	75 RH%
α-I	72	43	27	12
α-II	68	39	26	9
α-III	73	38	24	8
γ-I	68	41	24	9
γ-II	72	40	25	10
Q-I	53	-	-	-

**Table 5:** Eyring parameters for α-III.

	$V^{*}$ [m^3^]	ΔU [J mol^−1^]	${\dot{\epsilon }}_{0}$ [s^−1^]
*I*	9e-27	1e6	3e112
II	6e-27	3.2e5	3e48

**Table 6:** Eyring parameters for α-II.

	$V^{*}$ [m^3^]	ΔU [J mol^−1^]	${\dot{\epsilon }}_{0}$ [s^−1^]
*I*	9e-27	1e6	3e106
II	6e-27	3.2e5	8e47

**Table 7:** Eyring parameters for γ-II.

	$V^{*}$ [m^3^]	ΔU [J mol^−1^]	${\dot{\epsilon }}_{0}$ [s^−1^]
*I*	9e-27	1e6	1e123
II	1.9e-27	3e5	6e45
